# Epidemiologic features of the first MERS outbreak in Korea: focus on Pyeongtaek St. Mary’s Hospital

**DOI:** 10.4178/epih/e2015041

**Published:** 2015-09-17

**Authors:** Kyung Min Kim, Moran Ki, Sung-il Cho, Minki Sung, Jin Kwan Hong, Hae-Kwan Cheong, Jong-Hun Kim, Sang-Eun Lee, Changhwan Lee, Keon-Joo Lee, Yong-Shik Park, Seung Woo Kim, Bo Youl Choi

**Affiliations:** 1 Division of Epidemic Intelligence Service, Korea Centers for Disease Control and Prevention, Cheongju, Korea; 2 Department of Cancer Control and Policy, Graduate School of Cancer Science and Policy, National Cancer Center, Goyang, Korea; 3 Graduate School of Public Health and Institute of Health and Environment, Seoul National University, Seoul, Korea; 4 Department of Architectural Engineering, Sejong University, Seoul, Korea; 5 Department of HVAC & Firefighting Engineering, Gachon University College of Engineering, Seongnam, Korea; 6 Department of Social and Preventive Medicine, Sungkyunkwan University School of Medicine, Suwon, Korea; 7 Division of Malaria and Parasitic Diseases, Korea Centers for Disease Control and Prevention, Cheongju, Korea; 8 Department of Preventive Medicine, Hanyang University College of Medicine, Seoul, Korea

**Keywords:** Epidemiology, Nosocomial infection, Korea, Middle East Respiratory Syndrome coronavirus, Outbreak, Quarantine

## Abstract

**OBJECTIVES:**

This study investigated the epidemiologic features of the confirmed cases of Middle East Respiratory Syndrome (MERS) in Pyeongtaek St. Mary’s Hospital, where the outbreak first began, in order to identify lessons relevant for the prevention and control of future outbreaks.

**METHODS:**

The patients’ clinical symptoms and test results were collected from their medical records. The caregivers of patients were identified by phone calls.

**RESULTS:**

After patient zero (case #1) was admitted to Pyeongtaek St. Mary’s Hospital (May 15-May 17), an outbreak occurred, with 36 cases between May 18 and June 4, 2015. Six patients died (fatality rate, 16.7%). Twenty-six cases occurred in the first-generation, and 10 in the second-generation. The median incubation period was five days, while the median period from symptom onset to death was 12.5 days. While the total attack rate was 3.9%, the attack rate among inpatients was 7.6%, and the inpatients on the eighth floor, where patient zero was hospitalized, had an 18.6% attack rate. In contrast, caregivers and medical staff showed attack rates of 3.3% and 1.1%, respectively.

**CONCLUSIONS:**

The attack rates were higher than those of the previous outbreaks in other countries. The outbreak spread beyond Pyeongtaek St. Mary’s Hospital when four of the patients were moved to other hospitals without appropriate quarantine. The best method of preventing future outbreaks is to overcome the vulnerabilities observed in this outbreak, such as ward crowding, patient migration without appropriate data sharing, and the lack of an initial broad quarantine.

## INTRODUCTION

The Middle East Respiratory Syndrome coronavirus (MERS-CoV) [1] is a positive-sense RNA virus of the coronaviridae family that was first reported in Saudi Arabia in 2012. Information about its transmission pattern is still scant. Although MERS-CoV has been shown to have a fatality rate as high as 40% [2], no medication or vaccine with proven efficacy has yet been developed. Although the first case of MERS-CoV was reported in Saudi Arabia in October 2012, nosocomial infection with MERS-CoV was reported in Jordan as early as March 2012 [3]. Thereafter, MERS-CoV has caused outbreaks of various scales in hospitals, while community-acquired infections have remained extremely rare. Although most patients with MERS-CoV have been reported in the Middle East, both before and after the outbreak in the Republic of Korea (hereafter Korea) in May 2015, the outbreak in Korea was the largest in any country other than Saudi Arabia. This outbreak made MERS-CoV an important international health issue [4]. After the first outbreak in Pyeongtaek St. Mary’s Hospital, where the patient zero was hospitalized, the outbreak spread when infected patients moved to other hospitals, resulting in an unprecedentedly large outbreak in Korea [5]. In this study, we aimed to analyze the epidemiological features of the MERS cases in Pyeongtaek St. Mary’s Hospital, where the first outbreak of MERS-CoV in Korea took place, with the goal of identifying lessons capable of helping to prevent and/or control future outbreaks.

## MATERIALS AND METHODS

### Definitions

A confirmed MERS-CoV case was defined as a person with a definitive diagnosis of MERS based on laboratory tests, regardless of clinical symptoms. The first day of symptoms was the day when clinical symptoms related to MERS-CoV infection first occurred, including all non-specific symptoms such as fever, chills, shortness of breath, cough, sputum, sore throat, myalgia, diarrhea, nausea and vomiting.

The incubation period of a disease is the interval from exposure to the occurrence of symptoms. However, it was difficult to calculate the exact incubation period in cases where the date of exposure or the date of the onset of symptoms was unclear. When the exposure period was longer than two days, the incubation period was considered to extend from the midpoint of the possible exposure period to the first onset of symptoms.

Patient zero was defined as generation zero, and the patients infected by patient zero comprised the first-generation, followed by the second-generation and third-generation [6]. This study defined the first-generation as those who were exposed to patient zero and showed symptoms within a maximum of 14 days. The second- generation was defined as cases who were exposed to first-generation cases without exposure to patient zero, or who were exposed to patient zero but showed symptoms more than 14 days after exposure. Similarly to the second-generation, the third-generation was defined as cases who were exposed to the second- generation without exposure to the first-generation, or who were exposed to the first-generation but showed symptoms more than 14 days after being exposed. Some cases were exposed to both patient zero and the first-generation or both the first-generation and second-generation. In such cases, the cases were classified as belonging to the group with the higher chance of infection based on the epidemiologic investigation report.

### Subjects

Thirty-six confirmed cases were infected at Pyeongtaek St. Mary’s Hospital, where the MERS outbreak first occurred in Korea. The epidemiologic features of patient zero have been separately described (in press) and were not included in this analysis of Pyeongtaek St. Mary’s Hospital, beyond a short summary of his clinical history.

### Data collection

The epidemiologic features of the cases were collected from the epidemiologic investigation report that was drafted after inspection of the hospital and patient interviews. The epidemiologic investigation report contained the following information for each case: demographic details, MERS-CoV exposure history, underlying diseases, clinical history, the occurrence of symptoms related to MERS-CoV infection, contact history, and history of post-exposure quarantine. Moreover, closed-circuit television analysis data were used to identify the cases’ contact histories, while hospital visit records and data about underlying diseases were collected from the Health Insurance Review and Assessment Service. The patients’ medical records were reviewed for clinical symptoms and test results.

The list of patients, medical staff members, and caregivers (family members, health aides, or visitors of patients) who were exposed to MERS-CoV in Pyeongtaek St. Mary’s Hospital was provided by the hospital, and information about hospitalization dates and room numbers was extracted from this dataset. The denominators used to calculate the MERS-CoV attack rate were the number of hospitalized patients and medical staff on the eighth floor starting from May 15, when patient zero was admitted, to May 21, when the eighth floor of the hospital was shut down, and on the seventh floor from May 20, when the first-generation cases started to move to the seventh floor, to May 29, when the hospital was closed. The caregivers of exposed patients were identified via phone calls.

### Laboratory test

Sputum or tracheal aspiration specimens from the lower respiratory tract were used to diagnose MERS-CoV. When sputum specimens were unavailable, nasopharyngeal or oropharyngeal swab specimens were used instead. The collected specimens were tested in the Respiratory Virus Department of the Korea Centers for Disease Control and Prevention (KCDC). MERS-CoV was identified by two target genes (an upstream of MERS-CoV envelope protein gene and open reading frame 1a gene) using two real-time reverse transcriptase-polymerase chain reaction according to World Health Organization guidelines [7].

### Data analysis

Since the study involved a small sample size (36 cases, six of whom died), the log likelihood test was used to analyze correlations between cases’ demographic and clinical features and the fatality rate. A non-parametric test was performed for time intervals (duration between symptom onset and confirmation or discharge), because the data were not normally distributed. SPSS version 22.0 (IBM Corp., Armonk, NY, USA) was used for statistical analysis, and p-values<0.05 were considered to indicate statistical significance.

## RESULTS

### Characteristics of the outbreak

The first-generation of cases began to show symptoms on May 18, after patient zero was hospitalized at Pyeongtaek St. Mary’s Hospital from May 15 to May 17, 2015. These cases led to additional infections, resulting in a total of 36 confirmed cases by June 4. Since these infections were caused by exposure within the hospital, they were considered nosocomial infections. Neither inpatients on the fourth floor of the hospital nor outpatients were infected by MERS-CoV, and no MERS-CoV cases were found among medical staff members or hospital personnel other than the nurses on the seventh and eighth floors.

### Patient zero (case #1)

Patient zero was a 68-year-old man with hypertension, hyperlipidemia, and benign prostatic hypertrophy who often used an inhaler for asthma, although asthma did not impede his daily life. He visited Saudi Arabia and the United Arab Emirates while based out of Bahrain for business during the 11-day period from April 24 to May 4 and returned via Qatar. He had no contact with camels during his stay in the Middle East and never visited a local hospital. Although he met some local buyers in business meetings in Saudi Arabia, he did not remember interacting with anyone with respiratory symptoms.

After returning to Korea, he visited Asan Seoul Clinic on May 12, 14, and 15 for fever and coughing that started on May 11. He was admitted to Pyeongtaek St. Mary’s Hospital on the afternoon of May 15, and remained hospitalized until the morning of May 17. He visited the 365 Clinic in Seoul on May 17 and went to the emergency room of Samsung Medical Center in Seoul, but returned home because no beds were open. The next morning, May 18, he was admitted through the emergency room. Samsung Medical Center in Seoul suspected MERS-CoV and requested confirmation from the KCDC, who confirmed MERS-CoV on May 20. He was then transferred to the National Medical Center of Korea for treatment. His condition became aggravated, requiring treatment with mechanical ventilation and extracorporeal membrane oxygenation, but was found to have cleared the MERS-CoV infection by June 30. After rehabilitation treatment in the general ward until September 25, he was discharged.

### Epidemic curve

After the hospitalization of patient zero, 36 infections occurred at Pyeongtaek St. Mary’s Hospital between May 18 and June 4. The peak of the outbreak occurred in the first-generation on May 21, while the second-generation cases first became symptomatic on May 25. No new cases have occurred since the last case was identified on June 4.

**Figure 1.**
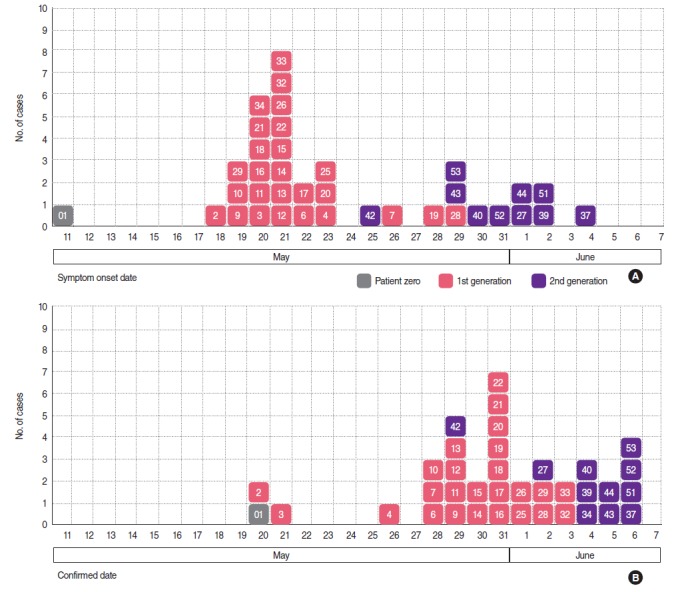
Epidemic curves of MERS outbreak at Pyeongtaek St. Mary’s Hospital, Korea. (A) Curve by symptom onset date, (B) curve by confirmed date.

Of the 36 cases, 26 belonged to the first-generation, and the other 10 appeared to belong to the second-generation. However, seven first-generation cases could have actually been second-generation cases, and five second-generation cases may have actually been third-generation cases (Figure 1, Appendix 1).

### Incubation period

**Figure 2.**
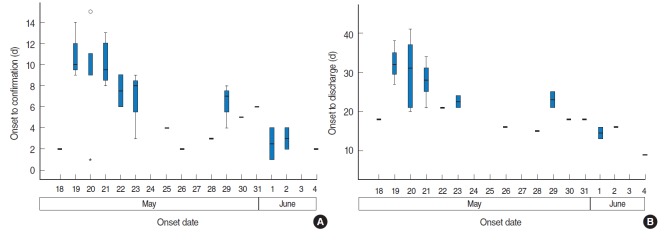
Duration from MERS symptom onset to confirmation (A) and discharge (B) by onset date, Pyeongtaek St. Mary’s Hospital, Korea.

**Figure 3.**
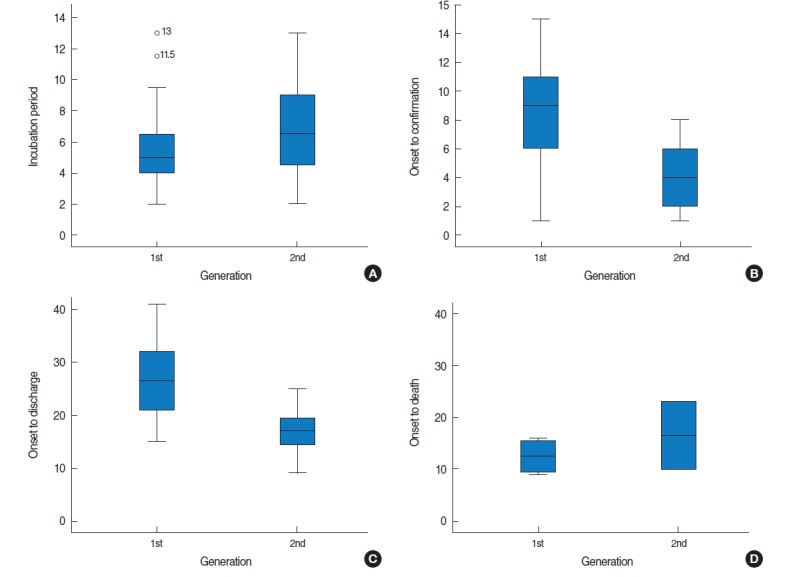
Boxplots for days of MERS outbreak by generation in Pyeongtaek St. Mary’s Hospital, Korea. (A) Incubation period (p = 0.227), (B) symptom onset to confirmation (p = 0.003), (C) symptom onset to discharge (p = 0.002), and (D) symptom onset to death (p = 0.533). The p-values were obtained by Mann-Whitney U test.

The median incubation period, calculated as the interval from the midpoint of the exposure period to the first onset of symptoms, was five days (range, 2 to 13 days). The incubation period increased as infection stage increased, but not to a statistically significant extent (p=0.227 by the Mann-Whitney U test) (Figures 2 and 3).

### Period between symptom onset and confirmation of MERS

The median duration from symptom onset to confirmation of MERS-CoV infection was eight days, ranging from the day of the onset of symptoms to 15 days. The median period for inpatients and medical staff was seven days, compared to nine days for the caregivers, although this difference was not statistically significant. The interval from the onset of symptoms to confirmation of the MERS decreased as the outbreak proceeded. Patients who were immediately subjected to quarantine, such as the wife of patient zero (case #2), the patient in the same room with patient zero (case #3), and the medical staff who treated patient zero (case #7) were subjected to tests immediately after the onset of symptoms and diagnosed within one to two days. Other patients who were quarantined before the onset of symptoms (cases #4, #37) were diagnosed rapidly. Much more time was required to diagnoses cases who experienced symptoms before May 29 and were not quarantined. The interval between symptom onset and diagnosis decreased as the infection stage increased, from nine days (range, 1 to 15 days) for the first-generation to four days (range, 1 to 8 days) for the second-generation. This was a statistically significant difference (p=0.003 by the Mann-Whitney U test) (Figures 2 and 3, Appendix 1).

### Attack rate

The transmission period during which patients with MERS-CoV were hospitalized at Pyeongtaek St. Mary’s Hospital was May 15 to May 29. Patient zero was hospitalized from May 15 to May 17; thereafter, first-generation and second-generation cases were hospitalized until May 29, when the hospital was shut down. During this period, a total of 929 people were exposed, of whom 36 were infected, resulting in an attack rate of 3.9%. During the transmission period, a total of 263 inpatients were present in the hospital, of whom 20 were ultimately diagnosed with MERS (attack rate, 7.6%). However, 13 of the 70 patients who were hospitalized on the eighth floor between May 15 and May 21 were confirmed to have been infected by MERS-CoV (attack rate, 18.6%). Thereafter, seven of the 148 inpatients of the seventh floor were infected from May 20 to May 29, during the period in which the inpatients on the eighth floor were moved to the seventh floor (attack rate, 4.7%). However, no MERS case occurred among the 45 patients on the seventh floor who were hospitalized only between May 15 and May 19. Thirteen of the 389 caregivers who were identified as being present on the seventh or eighth floors between May 15 and May 29 were confirmed to have been infected with MERS-CoV (attack rate, 3.3%). Three of the 277 medical staff members who were working during that period were diagnosed with MERS-CoV (attack rate, 1.1%). In contrast, two of 16 nurses on the eighth floor had confirmed MERS-CoV infections (attack rate, 12.5%), while one of the 20 nurses on the seventh floor had a confirmed MERS-CoV infection (attack rate, 5.0%). However, no nurses or medical staff members who were working on other floors or in outpatient departments were infected (Table 1).

### Demographic and clinical characteristics

The median age of the 36 cases of MERS-CoV infection (20 males [55.6%], 16 females [44.4%]) at Pyeongtaek St. Mary’s Hospital was 51 years (range, 24 to 79 years). Nineteen cases (52.8%) had underlying diseases, with a relatively higher prevalence among the men. Among the 36 cases, the proportion of inpatients, caregivers, and medical staff members was 55.6% (n=20), 36.1% (n=13), and 8.3% (n=3), respectively. One of 13 caregivers with a confirmed MERS-CoV infection was a visitor, and none were health aides. The symptoms of the 36 cases during the clinical course of MERS included fever (97.2%), coughing (55.6%), myalgia (47.2%), and sputum (44.4%), and the digestive symptoms included diarrhea (19.4%), nausea (11.1%), and vomiting (5.6%) (Table 2).

### Clinical outcomes and fatality rates

Thirty of the 36 cases recovered fully, but remaining six cases died, resulting in a 16.7% fatality rate. All of the deceased cases had underlying diseases, resulting in a fatality rate of 31.6% among the 19 cases with underlying diseases (p=0.003).

Cases in their seventies and fifties had 50% and 27.3% fatality rates, respectively (p=0.013). The fatality rates of males and females were 15.0% and 18.8%, respectively (p=0.765). Inpatients had a higher fatality rate (25.0%) than caregivers (7.7%), but this difference was not statistically significant (p=0.235). The fatality rates of the first-generation and second-generation cases were 15.4% and 33.3%, respectively (p=0.290).

The fatality rate of cases with pneumonia during the clinical course of MERS was 23.1% (p=0.876), and three of the five cases who required mechanical ventilation treatment died, showing a 60% fatality rate (p=0.014). All three cases who required treatment with extracorporeal membrane oxygenation died (p< 0.001), while the patient zero was not included in this analysis (Table 2).

### Period from the onset to discharge or death

The median period from the onset of clinical symptoms to discharge was 22.5 days (range, 9 to 41 days). This interval was 21, 21, and 26 days for inpatients, medical staff members, and caregivers, respectively (p=0.274 by the Kruskal-Wallis test). The period between symptom onset and discharge was 26.5 days (range, 15 to 41 days) for the first-generation and 17 days (range, 9 to 25 days) for the second-generation (p=0.002) (Figures 2 and 3).

The median intervals from confirmation of MERS to discharge were 17 days (range, 6 to 30 days) for the first-generation and 12.5 days (range, 5 to 17 days) for the second-generation, which was a statistically significant difference (p=0.048).

In the cases who died, the median duration from the onset of symptoms to death was 12.5 days (range, 9 to 23 days); in the first-generation, this interval was 12.5 days (range, 9 to 16 days), compared to 16.5 days in the second-generation (range, 10 to 23 days), but this difference was not statistically significant (p=0.533) (Figure 3).

## DISCUSSION

The 2015 outbreak of MERS-CoV in Korea began at Pyeongtaek St. Mary’s Hospital. After the hospitalization of patient zero from May 15 to May 17, 36 cases of MERS occurred during the 16-day period from May 18 to June 4. Six of these cases died, resulting in a fatality rate of 16.7%. The remaining 30 cases recovered and were discharged.

The median incubation period of the 36 cases was five days, similar to what has been reported in Saudi Arabia [3,8-10]. Although the median duration from symptom onset to confirmation of MERS-CoV infection was eight days, it became significantly shorter as the epidemic spread. In addition, the intervals from symptom onset to discharge and from diagnosis to discharge were significantly shorter for the second-generation cases than for the first-generation cases. These findings indicate that the confirmation of MERS-CoV infection in the first-generation of cases was initially relatively delayed, while the duration of treatment before discharge was higher. However, no significant difference in fatality rate by generation was observed, unlike the findings of a previous study in Saudi Arabia that found a lesser degree of severity in the second-generation [11]. Therefore, it seems that earlier confirmation of infection and treatment could reduce the overall duration of treatment, although it does not seem to be the case that the symptoms of the second-generation cases were more benign than those of the first-generation cases.

The most common clinical symptom was fever, as has been reported in previous studies. All cases without underlying diseases survived, whereas the fatality rate of cases with underlying diseases was 31.6%. Both of these fatality rates were lower than those reported in previous studies from Saudi Arabia, which reported fatality rates of approximately 10% for patients without underlying diseases and 41% for patients with underlying diseases [11-13]. However, this discrepancy may be due to differences in case composition between Saudi Arabia and Korea, so it would be necessary to compare our results with studies analyzing larger numbers of cases in order to draw any secure conclusions.

The attack rate of MERS-CoV in the entire cohort of exposed subjects was 3.9%, but that of inpatients on the eighth floor, who were in closest contact with patient zero, was 18.6%. Moreover, the attack rate among the medical staff members on the eighth floor was 12.5%, which was slightly higher than the attack rate among medical staff members (10%) in a previous report from Jordan [3].

The most important reason that the outbreak spread throughout Pyeongtaek St. Mary’s Hospital and beyond was the failure to implement a broad and strict quarantine in the early stage of the outbreak. Proper initial quarantine measures would have ended the outbreak with the 26 cases of the first-generation. However, the extent of the initial quarantine was limited to healthcare workers and those in the room where patient zero was hospitalized, and it did not include other inpatients or caregivers on the same floor. Although the 2014 KCDC guidelines for controlling MERS-CoV state that those who have close contact with confirmed cases as well as those who have casual contact, such as exposure to the confirmed cases or contaminated areas, must be quarantined or monitored carefully [14], these guidelines were not fully implemented. This was related to delays in the confirmation of MERS-CoV infections after the initial onset of symptoms, and the lack of a quarantine caused new outbreaks by allowing the movement of several cases to other hospitals. The second reason for the spread of MERS-CoV was overcrowding of the ward with families, who are in charge of patient care in Korea, and visitors [5]. In fact, a total of 389 family members and visitors were exposed to MERS-CoV in Pyeongtaek St. Mary’s Hospital, which was higher than the number of exposed inpatients (n=277). As a result, of the 26 cases in the first-generation, 12 were the family members of inpatients and one was a visitor who was infected in a single visit. These findings were consistent with the problems that have already been noted in the outbreak in Saudi Arabia: overcrowding, the late recognition of MERS-CoV infection, and inadequate infection control practices [15].

Various possible pathways exist for the transmission of MERS-CoV. It has been speculated that transmission occurred not only through direct transmission by patient zero, but also through indirect transmission via medical staff members through fomites. The modes of transmission, including hospital ward structures, the ventilation system, and other possible routes of aerosol transmission in Pyeongtaek St. Mary’s Hospital, will be the subject of a study that will appear in the near future.

The limitations of this study are as follows. Since this study encompassed only one hospital, it is difficult to generalize these results to the overall outbreak in Korea, which involved other hospitals. In addition, since the number of subjects was relatively small, a more detailed multivariate analysis of patient characteristics could not be performed. Nevertheless, the significance of this study lies in the fact that it examined MERS cases in the hospital where the outbreak began in Korea.

In conclusion, this study investigated the epidemiologic features of MERS-CoV in Pyeongtaek St. Mary’s Hospital, the origin of the MERS-CoV outbreak in Korea. A total of 36 cases, including 26 in the first-generation and 10 in the second-generation, were identified, of whom four were moved to other hospitals without appropriate quarantine, causing the nationwide MERS-CoV outbreak. The best method of preventing future outbreaks of new infectious diseases is overcoming the weak points identified here, including ward overcrowding, uncontrolled patient migration between hospitals without an appropriate data-sharing system, and the absence of a quarantine of those who were initially exposed.

## Figures and Tables

**Table 1.** Attack rate of MERS at Pyeongtaek St. Mary’s Hospital, Korea

	Total	Patients^1^				Caregivers^2^	Medical staff			
		Total	Eighth floor (May 15-21)	Seventh floor (May 20-29)	Seventh floor (May 15-19)		Total	Nurses on eighth floor	Nurses on seventh floor	Other
Subjects (n)	929	263	70	148	45	389	277	16	20	241
Confirmed cases (n)	36	20	13	7	0	13	3	2	1	0
Attack rate (%)	3.9	7.6	18.6	4.7	0.0	3.3	1.1	12.5	5.0	0

1 Patient zero (case #1) was admitted to the eighth floor from May 15 to May 17. Other MERS cases stayed on the eighth floor from May 15 to May 21 and on the seventh floor from May 20 to May 29.

2 Caregivers were defined as the family members, health aides, or visitors of patients.

**Table 2.** Characteristics and fatality rates of MERS cases at Pyeongtaek St. Mary’s Hospital, Korea

		n	%	Deaths (n)	Case fatality rate (%)	p-value^1^
Sex	Male	20	55.6	3	15.0	0.76
	Female	16	44.4	3	18.8	
Age (yr)	20-29	4	11.1	0	0.0	0.01^2^
	30-39	2	5.6	0	0.0	
	40-49	11	30.6	0	0.0	
	50-59	11	30.6	3	27.3	
	60-69	2	5.6	0	0.0	
	70-79	6	16.7	3	50.0	
Case type	Patient	20	55.6	5	25.0	0.23
	Caregivers	13	36.1	1	7.7	
	Medical staff	3	8.3	0	0.0	
Generation	First	26	72.2	4	15.4	0.74
	Second	10	27.8	2	20.0	
Underlying diseases	No	17	47.2	0	0.0	0.003
	Yes	19	52.8	6	31.6	
	Diabetes mellitus	13	36.1	4	30.8	0.09
	Malignancy	9	25.0	3	33.3	0.14
	Respiratory diseases^3^	6	16.7	3	50.0	0.03
	Cardiac diseases^4^	5	13.9	3	60.0	0.01
	Chronic kidney disease	3	8.3	1	33.3	0.46
Clinical symptoms	Fever	35	97.2	5	14.3	0.05
	Cough	20	55.6	4	20.0	0.54
	Sputum	16	44.4	3	18.8	0.76
	Shortness of breath	4	11.1	2	50.0	0.09
	Myalgia	17	47.2	1	5.9	0.09
	Nausea, vomiting	5	13.9	0	0.0	0.16
	Diarrhea	7	19.4	1	14.3	0.85
Disease course	Pneumonia	13	36.1	3	23.1	0.44
	Mechanical ventilation	5	13.9	3	60.0	0.006
	ECMO	3	8.3	3	100.0	<0.001
Total		36	100.0	6	16.7	

ECMO, extracorporeal membrane oxygenation.

1 Log likelihood ratio test.

2 Trend test.

3 Respiratory diseases were defined as including chronic obstructive pulmonary disease and asthma.

4 Cardiac diseases were defined as including ischemic heart disease, arrhythmia, and heart failure.

## Appendix 1.

Summary of epidemiologic characteristics of patients with MERS at Pyeongtaek St. Mary’s Hospital, Korea.
