# Supplementary material for: Epidemiologic features of the first MERS outbreak in Korea: focus on Pyeongtaek St. Mary’s Hospital
Source: Epidemiol Health. 2015 Sep 17;37:e2015041. doi: 10.4178/epih/e2015041 (PMC4652064; doi:10.4178/epih/e2015041)
Supplement: Supplementary file 1 [file epih-37-e2015041-supplementary.pdf]

한국의 첫번째 MERS-CoV 유행의 역학적 특징: 평택 성모 병원을 중심으로

Kyung Min Kim[1]* , Moran Ki[2]* , Sung-il Cho[3] , Minki Sung[4] , Jin Kwan Hong[5] , Hae-Kwan Cheong[6] , Jong-Hun Kim[6] , Sang-Eun Lee[7] , Changhwan Lee[1] , Keon-Joo Lee[1] , Yong-Shik Park[1] , Seung Woo Kim[1] , Bo Youl Choi[8]

[1] Division of Epidemic Intelligence Service, Korea Centers for Disease Control and Prevention, Cheongju;
[2] Department of Cancer Control and Policy, Graduate School of Cancer Science and Policy, National Cancer Center, Goyang;
[3] Graduate School of Public Health and Institute of Health and Environment, Seoul National University, Seoul;
[4] Department of Architectural Engineering, Sejong University, Seoul;
[5] Department of HVAC & Firefighting Engineering, Gachon University College of Engineering, Seongnam;
[6] Department of Social and Preventive Medicine, Sungkyunkwan University School of Medicine, Suwon;
[7] Division of Malaria and Parasitic Diseases, Korea Centers for Disease Control and Prevention, Cheongju;
[8] Department of Preventive Medicine, Hanyang University College of Medicine, Seoul, Korea

Abstract

목적: 처음 MERS의 유행이 시작된 평택 성모병원 확진자들의 역학적 특성을 통해서 기존에 알려진 유행들과의 차이점을 비교 분석하여 향후 MERS 예방과 관리에 도움을 주고자 하였다.

방법: 환자의 역학적 특징은 역학조사서를 기반으로 하였고, 병원 방문기록과 기저질환은 건강보험심사평가원의 자료를 확인하여 보충하였다. 환자의 임상적 증상과 검사 결과는 병원의 의무기록을 참고하였다. 환자의 보호자와 방문객은 각 환자 또는 보호자에게 전화 조사하여 확인하였다.

결과: 1번 환자(Patient zero, #1 case)가 평택성모병원에 입원(5월 15일부터 17일)한 이후 5월 18일부터 6월 4일까지 17일동안 36명의 환자가 발생하였고 이중에 6명이 사망하였다 (치명률 16.7%). 전체 환자 36명 중에 1차 감염(1st generation) 환자는 총 26명이고, 2차 감염으로 보이는 환자는 10명이었다. 잠복기 중위수는 5일(2-13일)이었고, 증상 발생에서 사망 중위수는 12.5일(9-23일)로 나타났다. 발병률은 3.9%이었으나 입원 환자 중에서는 7.6%이었고, 특히 1번 환자가 입원하였던 8층에서 15일부터 21일까지 입원하였던 환자들 중에서는 18.6%의 발병률을 보였다. 보호자는 3.3%, 의료진은 1.1%의 발병률을

나타내었다.

결론: 평택성모병원에서의 메르스 유행의 발병률은 기존에 알려진 것보다 높았다. 한국의 메르스 유행은 평택성모병원에서 감염된 4명의 환자가 적절히 격리되지 않은 상태에서 다른 병원으로 이동하면서 확산되었다. 초기에 광범위한 방역 부족, 적절한 정보공유 없이 환자들이 병원간에 이동하는 것, 혼잡한 병동 문제 등을 포함한 한국의 감염병 전파 취약점을 극복하는 것만이 향후 새로운 감염병 유행을 막을 수 있는 최선의 방법이 될 것이다.

Key words: Epidemiology, Nosocomial infections, Korea, Middle East Respiratory Syndrome coronavirus, Outbreak, Quarantine.

Introduction

메르스바이러스(Middle East Respiratory Syndrome Coronavirus, MERS-CoV)[1]는 Coronaviridae에 속한 양성극성의 RNA 바이러스로 2012년 사우디아라비아에서 처음 보고되어 아직 바이러스의 전파양상에 대한 정보가 매우 부족한 실정이다. 또한 메르스는 40%에 달하는 높은 치명률을 보임에도[2] 불구하고 아직 효과가 검증된 치료제와 백신은 개발되어 있지 않은 상황이다. 2012년 10월에 사우디아라비아에서 첫 환자가 보고되었으나, 메르스바이러스가 세상에 알려진 이후 2012년 3월에 이미 요르단에서 메르스 바이러스에 의한 병원 감염이 있었던 것이 보고되었다[3]. 이후 메르스 바이러스는 지역사회 감염은 매우 드물지만 병원에서는 크고 작은 유행을 일으키는 것으로 보고되고 있다. 주로 중동지역에서 대부분의 환자가 발생했으나 2015년 5월에 한국에서 유행이 시작되면서 한국의 유행은 사우디아라비아 이외의 국가에서 가장 큰 유행 사례로

남게 되었다. 이 유행은 전세계적으로도 메르스가 중요한 국제보건 이슈로 떠오르게 되는 계기가 되기도 하였다[4]. 한국의 큰 유행은 첫번째 환자가 입원하였던 평택 성모병원에서 유행이 발생하고 이곳에서 감염된 환자들이 다른 병원으로 이동하면서 병원에서 병원으로 유행이 확산되어 전례 없는 큰 유행을 만들어 냈다[5]. 저자들은 한국 최초 메르스 유행이 발생한 평택 성모 병원을 중심으로 환자의 역학적 특성을 분석하여 향후 질병 예방 및 관리에 활용하고자 한다.

Methods

(정의)

메르스 확진자는 임상증상과 무관하게 검사실 확진이 된 경우로만 정의하였다. 증상 시작일은 메르스 감염과 연관되어 처음 발생한 임상적 증상이 시작된 날로 정의하였다. 메르스 감염과 연관된 증상으로는 발열, 오한, 숨가쁨, 기침, 가래, 목아픔, 근육통, 설사, 오심과 구토 등 비특이적 증상을 모두 포함하였다.

잠복기는 노출 후 증상발생까지의 기간이다. 그런데 노출이 불명확하거나 기간이 2일이상 길 경우에 정확한 날짜를 지정하기 어렵다. 이 연구에서는 노출 기간의 중간 시점부터 최초 증상 발생까지의 기간으로 계산하였다.

감염 전파 단계는 1번환자가 generation zero, 1번환자에 의한 감염자는 1차 감염(1st generation), 이후부터 2차 감염(2nd generation), 3차 감염 (3rd generation)으로 정의하였다 [6]. 특히 이 연구에서 1차 감염은 1번환자에게 노출이 되었고 최대 잠복기 14일이내 발생한 경우로 정의하였고, 2차 감염은 1차 감염된 환자들에게 노출되었으면서, 1번환자에게는 노출이 없었거나, 1번환자 노

출이 있었으나 최대잠복기인 14일 이후에 발생한 환자로 정의하였다. 3차 감염은 2차 감염과 같은 방식으로 2차 감염자에게 노출되었으면서, 1차감염자에게는 노출이 없었거나, 또는 1차감염자에게 노출이 있었지만 최대잠복기인 14일 이후에 발생한 환자로 정의하였다. 이 연구에서는 1차 감염과 2차 감염의 가능성이 모두 있는 경우와, 2차와 3차 감염의 가능성이 모두 있는 환자들도 있었다. 이들은 역학조사서를 기준으로 가능성이 좀더 높은 쪽으로 분류하여 분석하였다.

(대상자)

한국에서 발생한 MERS 유행의 첫 유행지역인 평택성모병원에서 감염된 36명의 확진 환자들을 대상으로 하였다. 1번환자의 특성은 별도의 논문에서 기술할 예정이어서, 이번 평택 성모 감염자 분석에는 포함하지 않았다.

(자료 수집)

환자의 역학적 특징은 역학조사관이 환자를 면담하면서 조사하여 기록한 역학조사서를 기반으로 하였다. 역학조사서는 환자의 인적사항과 메르스 노출력, 기저질환, 임상 과거력, 이번 메르스 질환과 관련된 증상 발현, 접촉력, 접촉자에 대한 방역 시행내용 등을 포함하고 있다. 또한 사망과 퇴원 등의 추후 변동사항에 대해서는 이후에 보완이 이루어졌다. 추가로 환자의 접촉력을 확인하기 위해서 CCTV 분석 자료를 참고하였고, 환자의 병원 방문기록과 기저질환은 건강보험심사평가원의 자료를 확인하여 보충하였다. 환자의 임상적 증상과 검사 결과는 병원의 의무기록을 참고하였다.

평택성모병원에서 메르스에 노출된 환자들과 의료진, 보호자의 명단은 해당 병원에서 제공한 것을 이용하여 입원일과 병실을 확인하였다. 메르스 발병률을 구하기

위하여 병원의 8층은 첫환자가 입원하였던 15일부터 8층을 폐쇄한 21일까지를, 7층은 8층 환자가 내려오기 시작한 20일부터 병원을 폐쇄한 29일까지 입원한 환자와 해당 일에 근무한 의료진을 분모로 하였다. 환자의 보호자와 방문객은 각 환자 또는 보호자에게 전화 조사하여 확인하였다.

(실험실 검사방법)

메르스 진단을 위한 하기도 호흡기 검체는 객담이나 tracheal aspiration specimen을 이용하였다. 객담 검체를 이용하기 어려운 경우에는 nasopharyngeal or oropharyngeal swab 검체를 이용하였다. 수집된 검체는 질병관리본부의 호흡기바이러스과에서 검사하였다. MERS-CoV 확인은 WHO의 가이드라인을 따라 rRT-PCR(Real-time reverse-transcriptase-polymerase-chain reaction)로 두 개의 표적유전자 (an upstream of MERS-CoV envelope protein gene and open reading frame 1a Gene)를 이용하였다[7].

(자료분석방법)

전체 분석 대상자가 많지 않아(36명 발생, 6명 사망) 환자의 인구학적, 임상적 특징과 치명률의 연관성에 대한 분석은 Log likelihood test를 이용하였다. 임상증상에서 확진, 퇴원까지의 기간 등에 대한 그룹별 비교 분석은 정규분포를 하지않아 비모수 검정을 시행하였다. 통계분석은 SPSS Statistics Software, version 22.0 (IBM Corp., Armonk, NY, USA) 을 활용하였고, 통계적 유의성은 유의수준 5% 미만을 기준으로 하였다.

Results

(유행 양상: Characteristics of the outbreak)

평택성모병원에 1번환자가 입원한 2015년 5월 15일부터 5월 17일 사이에 노출된 같은 병동의 환자들과 보호자, 의료진의 1차 감염환자들의 증상이 18일부터 시작되었고, 이들 환자들에 의한 추가 감염으로 인하여 6월 4일까지 총 36명의 확진자가 발생하였다. 감염은 모두 병원내에서 노출된 사람들로 한정되어 병원 감염이라고 할 수 있다. 또한 병원의 4층에 입원하였던 환자나 외래환자 중에서는 메르스 환자 발생이 없었으며 7, 8층 간호사 이외의 의료진이나 병원 관계자 중에서도 메르스 환자 발생은 없었다(Figure 1).

(1번환자: patient zero, case #1)

1번환자는 68세 남자로 고혈압, 고지혈증, 전립선비대증으로 약물 복용 중이었고, 천식으로 가끔 흡입기 사용은 했으나, 일상생활에 장애는 없었다. 4월 24일부터 5월 4일까지로 11일동안 바레인을 중심으로 사우디아라비아와 아랍에미리트를 사업차 방문하였고, 카타르를 거쳐 출입국하였다. 중동에 있는 동안 낙타와 접촉한 적이 없고, 현지에서 병원을 방문한 적도 없었다. 사우디 아라비아에서 사업 미팅을 하면서 현지바이어들을 만났으나 호흡기 증상이 있었던 사람은 없는 것으로 기억하였다. 한국에 들어온 이후 5월 11일에 발열, 기침 등이 나타나, 5월 12일, 14일, 15일에 아산에 있는 의원(아산 서울의원)을 방문하였다. 평택성모병원에는 5월 15일 오후-17일 오전까지 입원하였고, 5월 17일에 서울로 이동하였다. 5/17일에 서울의 365의원에 들렀다가 삼성서울병원 응급실로 갔으나 입

원실이 없어 다시 집으로 돌아온 후 다음날 18일 오전에 응급실 통하여 입원하게 되었다. 삼성서울병원에서 메르스 의심하에 질병관리본부에 요청하여 5/20일에 메르스 확진을 받고 국립중앙의료원으로 전원되어 치료를 받았다. 한때는 인공호흡기 치료와 에크모 치료까지 받으면서 매우 위중했으나 6월 30일 메르스 바이러스는 음전되었고, 일반 병동에서 재활 치료 후 9월 25일 퇴원하였다.

(유행곡선: Epidemic curve)

Patient zero (#1 case) 입원 이후 평택성모 병원에서 5월 18일부터 6월 4일까지 19일 동안 36명의 환자가 발생하였다. 5월 21일에 1차 감염(1st generation) 환자에 인한 첫번째 유행 정점이 나타났고 2차 감염자는 5월 25일부터 발생하기 시작하였다. 6월 4일에 마지막 환자가 발생한 이후 추가환자는 없었다.

전체 환자 36명 중에 1차 감염 환자는 총 26명이고, 2차 감염으로 보이는 환자는 10명이었다. 그런데 1차 감염자 중에서 2차 감염의 가능성이 있는 환자는 7명이었고, 2차 감염자 중에는 3차 감염의 가능성이 있는 환자가 5명 있었다 (Figure 1, Appendix 1).

(잠복기: Incubation period)

노출 기간의 중간 시점부터 최초 증상 발생까지의 기간으로 계산한 잠복기의 중위수는 5일 (2일-13일)로 나타났다. 감염차수에 따라 잠복기가 길어지는 것으로 나타났으나 통계적으로 유의하지는 않았다 ($p=0.227$ by Mann-Whitney U test) (Figures 2 and 3).

(증상 발생부터 확진까지의 기간: Period between symptom onset and confirmation of MERS)

증상 발생부터 확진까지의 기간 중위수는 8일이었으나 환자에 따라 증상 발생 당일에서 15일까지의 편차를 보였다. 환자유형별로 보면 병원에 입원했던 환자와 의료진은 중위수가 7일, 보호자/방문객은 9일로 차이가 있으나 통계적으로 유의하지는 않았다. 증상발생일에 따라서 유행이 진행되면서 점차 증상발생부터 확진까지의 기간이 감소되는 것을 확인할 수 있었다. 또한 처음부터 격리대상자로 선정되었던 1번환자의 부인(#2), 같은 병실에 있던 환자(#3), 의료진(#7)등은 증상 발생 후 바로 검사가 진행되어 하루 이틀만에 진단이 되었다. 또한 증상발생 전에 격리를 시행하고 있던 사람(#4, #37)도 진단이 빠르게 이루어졌다. 하지만 5월 29일 이전에 증상이 발생하였으나 격리되지 않았던 환자들은 진단이 매우 늦어졌다. 감염차수에 따라서도 1차 감염 9(1-15)일, 2차 감염 4(1-8)일로 감소하는 경향을 보였으며 통계적으로도 유의하였다 (p = 0.003 by Mann-Whitney U test) (Figures 2 and 3, Appendix 1).

(발병률: Attack rate)

평택성모병원에 메르스 환자가 있었던 전파 가능 기간(transmission period)은 5월 15일부터 29일까지이다. 15일부터 17일까지는 1번환자가 입원하고 있었고, 이후 1차 감염된 환자들과 이들에 의하여 감염된 2차 환자들이 이 병원이 29일에 폐쇄될때까지 입원하고 있었다. 이 기간동안 노출된 사람은 총 929명이었고 이중에 36명이 감염되어 발병률은 3.9%이었다. 노출 기간 동안 입원하고 있던

환자는 총 263명이었고, 이 중에 20명이 확진을 받아 발병률이 7.6%이었다. 그런데 8층에 15일부터 21일까지 입원하였던 70명 중에서는 13명이 확진되어 18.6%의 발병률을 보였고, 이후 8층의 환자들이 7층으로 전원하기 시작한 5월 20일부터 29일까지 7층에 입원하고 있었던 환자 148명 중에서는 7명이 감염되어 4.7%의 발병률을 보였다. 그러나 7층에 15일부터 19일까지만 입원하였던 환자 45명 중에서는 감염자가 없었다. 15일부터 29일 사이에 해당 병원의 7층 또는 8층에 있었던 것이 확인된 보호자(간병인 또는 방문객) 389명 중에서는 13명이 확진자로 나타나서 3.3%의 발병률을 보였다. 같은 기간에 근무한 의료진은 총 277명 중 3명이 확진되어서 1.1%의 발병률을 나타내었다. 그러나 8층의 간호사 16명 중에서는 2명이 확진을 받아서 12.5%의 발병률을 보였고, 7층 간호사 20명 중에서는 1명이 확진을 받아서 5.0%의 발병률을 보였다. 그 외 다른 층의 병동이나 외래에서 근무하였던 간호사 중에서는 감염자가 없었다. 또한 간호사 이외의 의료진에서도 감염자가 없었다 (Table 1).

(인구학적, 임상적 특징: Demographic and clinical characteristics)

평택성모병원에서 감염된 36명의 연령 중위수는 51세 (24-79)이고, 남자가 20명 (55.6%), 여자가 16명 (44.4%) 이었다. 기저질환을 가지고 있었던 환자는 19명 (52.8%)이었고 기저질환자 비율은 남자에서 조금 높았다. 입원 중이던 환자가 55.6% (n=20), 보호자가 36.1% (n=13), 의료진이 8.3% (n=3) 이었다. 확진된 보호자 13명 중에 1명은 방문객이었고 간병인은 없었다.

확진자 36명의 임상 경과 중에 나타났던 증상은 발열 97.2%, 기침 55.6%, 근육

통 47.2%, 가래 44.4%의 순서이고, 소화기 증상은 설사 19.4%, 오심 11.1%, 구토 5.6%로 나타났다 (Table 2).

(임상결과와 치명률: Clinical outcomes and case fatality rate)

확진자 36명 중 30명은 완치되었으나 6명은 사망하여 치명률은 16.7%를 나타내었다. 그런데 사망자는 모두 기저질환자에서 발생하여 기저질환자의 치명률은 31.6% (6/19명)로 나타났다 ($p = 0.003$).

연령별로는 70대에서 50%, 50대에서 27.3%로 나타났다 ($p=0.013$). 성별로는 남자 15.0%, 여자 18.8%로 나타났다 ($p=0.765$). 확진자 특성별로 보면 입원치료 중이던 환자의 치명률이 25.0%로 가장 높았다 ($p=0.235$). 감염 차수 별로 보면 1차가 15.4%, 2차가 33.3%이었다 ($p=0.290$).

임상경과중에 폐렴이 있었던 환자의 치명률은 23.1%이었고 ($p=0.876$), 인공호흡기 치료를 받았던 5명중에 3명이 사망하여 60%의 치명률을 보였다 ($p=0.014$). ECMO치료를 받았던 3명은 모두 사망하였다 ($p<0.001$) (Table 2).

(증상발생부터 퇴원 또는 사망까지의 기간: Period from the onset to discharge or death)

퇴원 환자 30명의 임상증상 발생에서 퇴원까지의 기간 중위수는 22.5일이었으나 환자에 따라 증상 발생 9일째에서 41일까지의 편차를 보였다. 환자유형별로 보면 입원중이던 환자는 21일, 의료진 21일, 보호자/방문객은 26일로 차이가 있으나 통계적으로 유의하지는 않았다 ($p=0.274$ by the Kruskal-Wallis test). 감염차수에

따라서는 1차 26.5(15-41)일, 2차 17(9-25)일로 차이가 있었으며 통계적으로도 유의한 경향을 보였다 (p=0.002) (Figures 2 and 3).

확진에서 퇴원까지의 기간 중위수는 감염차수에 따라서 1차 17(6-30)일, 2차 12.5(5-17)일로 차이가 있었으며 통계적으로도 유의한 경향을 보였다 (p=0.048).

메르스 증상 발생에서 사망까지의 기간은 중위수 12.5(9-23)일로 나타났다. 감염차수에 따라서는 1차 12.5(9-16)일, 2차 16.5(10-23)일로 차이가 있었으나 통계적으로는 유의하지 않았다 (p=0.533) (Figure 3).

Discussion

2015년 한국 메르스 유행은 평택성모병원의 유행에서 시작되었다. 1번환자가 5월 15일부터 17일까지 입원한 이후 5월 18일부터 6월 2일까지 15일동안 36명의 환자가 발생하였고 이중에 6명이 사망하였다 (치명률 16.7%). 30명은 모두 완치되어 퇴원하였다.

36명의 잠복기 중위수는 5일로 사우디 아라비아와 비슷하였다[3,8-10]. 증상 발생 후 확진까지의 기간 중위수는 8일이었으나 초기에 비해서 유행이 진행되면서 점차 유의하게 짧아졌다. 또한 증상 발생 후 퇴원까지의 기간이나, 확진 후 퇴원까지의 기간을 보면 1차 감염에 비하여 2차 감염에서 유의하게 짧아졌다. 이는 초기에 감염된 1차 감염자들의 경우에 상대적으로 확진이 늦어졌고, 또한 퇴원까지의 치료기간도 길어졌음을 의미한다. 그러나 치명률은 감염 차수에 따라 유의한 차이가 없었다. 이는 기존에 사우디아라비아에서 나온 연구 결과에서 2차 감염에서 중증도가 더 낮다는 결과와는 다르다[11]. 따라서 2차 감염이 1차 감염보다 증

상이 미약하다기 보다는 빠른 확진과 치료가 시행되어 전체적인 치료기간이 감소되었을 것으로 보인다.

임상적으로 가장 흔한 증상은 열이었고 이는 기존의 연구결과와 같았다. 치명률은 기저질환이 없는 경우에는 사망자가 없었고, 기저질환이 있었던 환자 중에서는 31.6%로 나타나 기존의 사우디아라비아 보고 (약 10%와 41%)에 비하여 낮았다 [11-13]. 그러나 이러한 차이는 환자 구성의 차이가 국가별로 다르므로 더 많은 환자들을 대상으로 분석하여 비교하는 것이 필요하다.

메르스 발병률은 전체 노출자에서는 3.9%이었으나 가장 직접적으로 노출이 되었던 8층의 입원환자들에서는 18.6%로 매우 높았다. 또한 8층의 의료진에서도 12.5%의 발병률을 보여 이전 요르단에서 보고한 의료진 발병률 10% [3] 보다 조금 높게 나타났다.

평택성모병원에서의 유행 확산은 초기에 접촉자들을 광범위하고 철저하게 격리하고 관리하지 못한 것이 가장 큰 원인이었다. 초기에 격리 조치가 적절히 되었다면 1차 감염자 26명으로 유행이 끝났을 수 있었을 것이다. 하지만 접촉자의 범위를 의료진과 1번환자가 있었던 병실 한곳으로 한정함으로써 동일 병동 내 다른 병실에 있었던 환자 및 보호자, 방문객들이 격리대상에 포함되지 않았다. 2014년도에 발간된 한국의 메르스 관리지침에서도 확진환자와 밀접하게 접촉한 사람과, 확진자나 오염된 환경에 노출된 일상적 접촉을 한 사람에 대해서도 자가격리 또는 능동감시를 하도록 되어있지만[14] 지침에 따른 접촉자 격리가 충실히 이행되지 않았다. 이는 초기 증상 발생 후 확진까지의 기간이 길어진 원인과 관련이 있고, 이 병원에서 여러 환자들이 다른 병원으로 이동하여 새로운 유행을 일으킨 계기가 되었다. 또한 입원

병동이 가족과 방문객 들로 인하여 매우 혼잡하였던 것이 유행을 크게 만든 두번째 원인이었다[5]. 실제 평택성모병원에서 메르스에 노출된 환자가족, 방문객은 모두 389명으로 이는 노출된 입원환자 277명보다 많았다. 그 결과 1차 감염된 26명 중에서 환자의 가족이 12명이었고, 단순 1회 방문 후 감염된 방문객도 1명 있었다. 그런데 이러한 문제들은 사우디 아라비아의 유행에서도 이미 지적되었던 문제들 즉, overcrowding, late recognition of MERS-CoV cases, and inadequate infection control practices 와 같다 [15].

감염전파 경로에 대해서는 여러가지 가능성이 있을 수 있다. 1번환자에 의한 직접전파뿐 아니라 의료진에 의한 간접전파(바이러스에 오염된 매개물에 의한 전파)도 일어났을 것으로 추측된다. 그리고 평택성모병원에서의 병실 구조와 환기구조, 바이러스 확산 가능성 등에 대해서는 추가 연구를 통하여 발표할 예정이다.

이 연구의 제한점은 다음과 같다. 이번 한국의 메르스 유행 전체 중에서 한 병원만의 결과이므로 이를 한국 유행 전체로 일반화 하기는 어렵다. 또한 대상자 수가 적어 환자 특성별로 깊이 있는 분석과 다변량 분석을 시행하지는 못하였다. 하지만 한국 유행의 시작이 되었던 첫 병원의 유행사례라는 점에서 그 의의가 있다.

결론적으로 한국의 메르스 유행 첫 진원지였던 평택성모병원에서 1차감염 26명, 2차 감염 10명으로 총 36명의 환자가 발생하였고, 이들 중에 4명이 적절한 격리 조치를 받지 않은 상태에서 다른 병원으로 이동하면서 메르스 유행이 전국으로 확산하게 되는 원인이 되었던 것으로 나타났다. 향후 새로운 감염병 유행을 막기 위해서는 이번에 나타난 병원 감염 취약점 (초기 접촉자 방역을 철저히 하지 못한 것, 병원간 적절한 자료 공유 시스템 없이 환자들이 이동한 것, 혼잡한 병동 문제 등)을

잘 극복해야만 할 것이다.

참고문헌

1. de Groot RJ, Baker SC, Baric RS, Brown CS, Drosten C, Enjuanes L, et al. Middle East respiratory syndrome coronavirus (MERS-CoV): announcement of the Coronavirus Study Group. J Virol 2013;87:7790-7792.
2. Hui DS, Memish ZA, Zumla A. Severe acute respiratory syndrome vs. the Middle East respiratory syndrome. Curr Opin Pulm Med 2014;20:233-241.
3. Al-Abdallat MM, Payne DC, Alqasrawi S, Rha B, Tohme RA, Abedi GR, et al. Hospital-associated outbreak of Middle East respiratory syndrome coronavirus: a serologic, epidemiologic, and clinical description. Clin Infect Dis 2014;59:1225-1233.
4. WHO. Global Alert and Response. Coronavirus infections. Available from: http://www.who.int/csr/don/archive/disease/coronavirus_infections/en/.
5. Ki M. 2015 MERS outbreak in Korea: Hospital-to-Hospital Transmission. Epidemiol Health 2015.
6. Farrington P. Modelling Epidemics. Oxford: The Open University; 2003, p13.
7. Corman VM, Eckerle I, Bleicker T, Zaki A, Landt O, Eschbach-Bludau M, et al. Detection of a novel human coronavirus by real-time reverse-transcription polymerase chain reaction. Euro Surveill 2012;17.
8. Assiri A, McGeer A, Perl TM, Price CS, Al Rabeeah AA, Cummings DA, et al. Hospital outbreak of Middle East respiratory syndrome coronavirus. N Engl J Med 2013;369:407-416.
9. Drosten C, Muth D, Corman VM, Hussain R, Al Masri M, HajOmar W, et al. An observational, laboratory-based study of outbreaks of middle East respiratory syndrome coronavirus in Jeddah and Riyadh, kingdom of Saudi Arabia, 2014. Clin Infect Dis 2015;60:369-377.
10. Memish ZA, Zumla AI, Al-Hakeem RF, Al-Rabeeah AA, Stephens GM. Family cluster of Middle East respiratory syndrome coronavirus infections. N Engl J Med 2013;368:2487-2494.
11. Memish ZA, Al-Tawfiq JA, Makhdoom HQ, Al-Rabeeah AA, Assiri A, Alhakeem RF, et al. Screening for Middle East respiratory syndrome coronavirus infection in hospital patients and their healthcare worker and family contacts: a prospective descriptive study. Clin Microbiol Infect 2014;20:469-474.
12. Drosten C, Meyer B, Muller MA, Corman VM, Al-Masri M, Hossain R, et al. Transmission of MERS-coronavirus in household contacts. N Engl J Med 2014;371:828-835.

13. Memish ZA, Zumla AI, Assiri A. Middle East respiratory syndrome coronavirus infections in health care workers. N Engl J Med 2013;369:884-886.
14. Korea Centers for Disease Control and Prevention (KCDC). Guideline for management of MERS. Korea Centers for Disease Control and Prevention; 2014, p22-23 (Korean).
15. Al-Tawfiq JA, Perl TM. Middle East respiratory syndrome coronavirus in healthcare settings. Curr Opin Infect Dis 2015;28:392-396.
